# Supplementary material for: PARP‐1 regulates DNA repair factor availability
Source: EMBO Mol Med. 2018 Nov 22;10(12):e8816. doi: 10.15252/emmm.201708816 (PMC6284389; doi:10.15252/emmm.201708816)
Supplement: Supplementary file 1 — Appendix [file EMMM-10-e8816-s001.pdf]

Appendix Table of Contents

pp2-9	Appendix Figures S1-S8
p10	Appendix Table S1

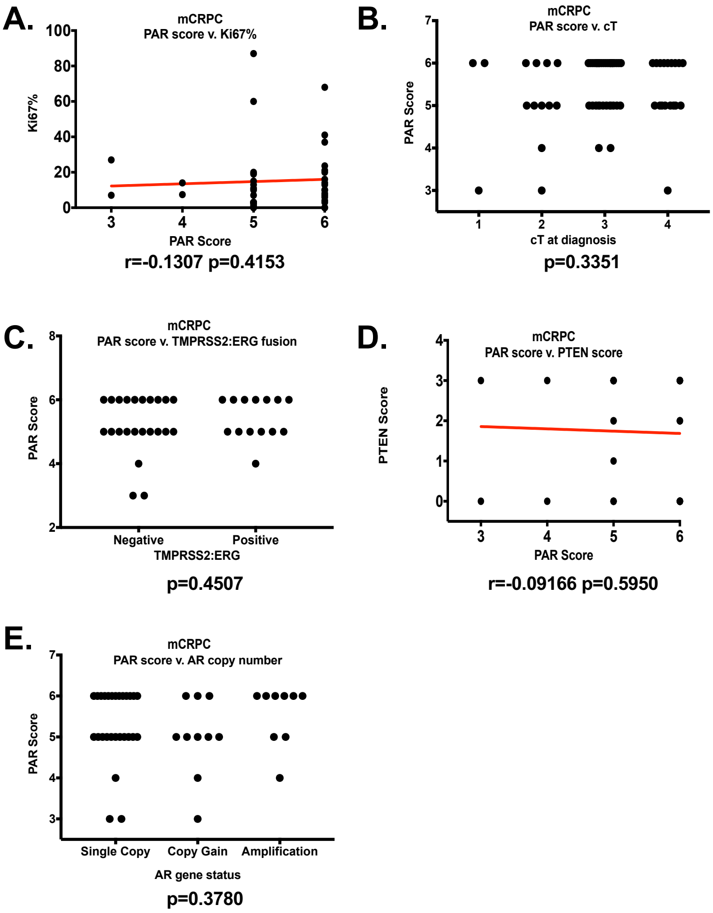

Appendix Figure S1. PARP-1 enzymatic activity is not associated with clinical parameters or molecular alterations that are frequent in PCa. PAR IHC score data from mCRPC specimens as described in Figure 1 was compared to **(A)** Ki67% positivity, **(B)** cT at diagnosis, **(C)** TMPRSS2:ERG fusion status, **(D)** PTEN IHC score, and **(E)** *AR* copy number status. Statistical analyses (r and p values) were derived by Spearman correlation.

A.

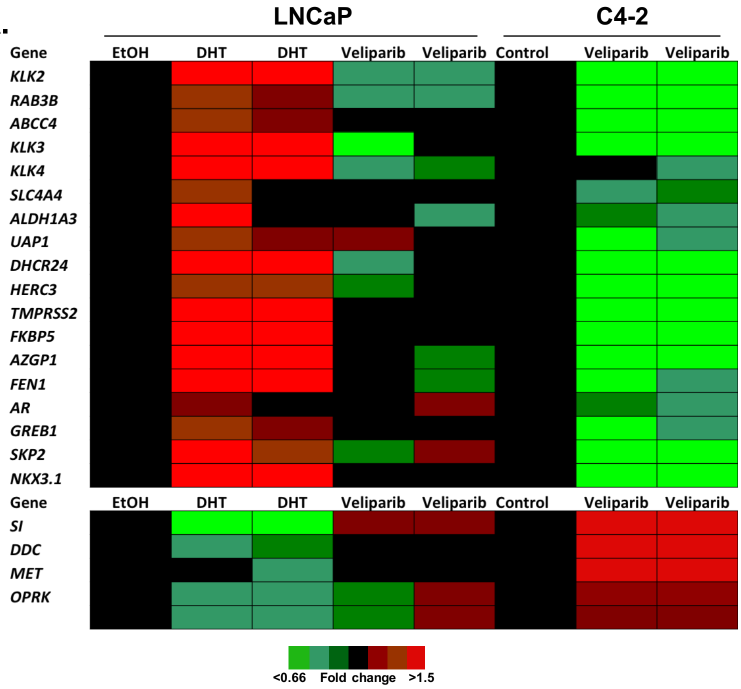

B.

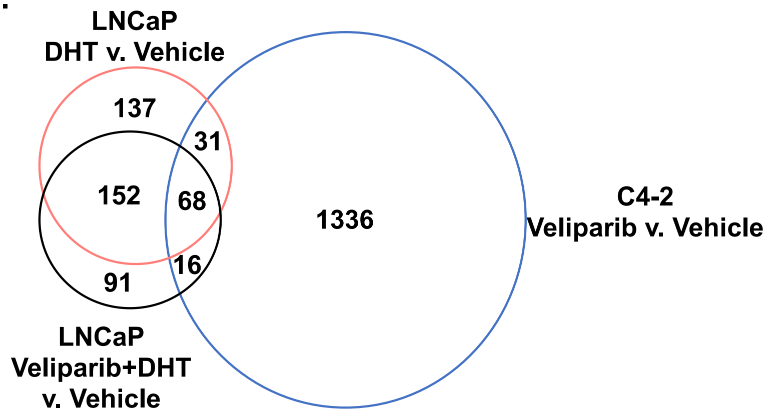

Appendix Figure S2. PARP-1 regulates overlapping and distinct transcriptional programs in HT-sensitive PCa CRPC cells. (A) Previously defined androgen/AR regulated genes were used to examine the effect of PARPi in the data generated in Figure 2. **(B)** Genes found to be significantly different p<0.05 and >1.5 fold change) in the indicated cell lines under the conditions described are depicted. DHT=dihydrotestosterone.

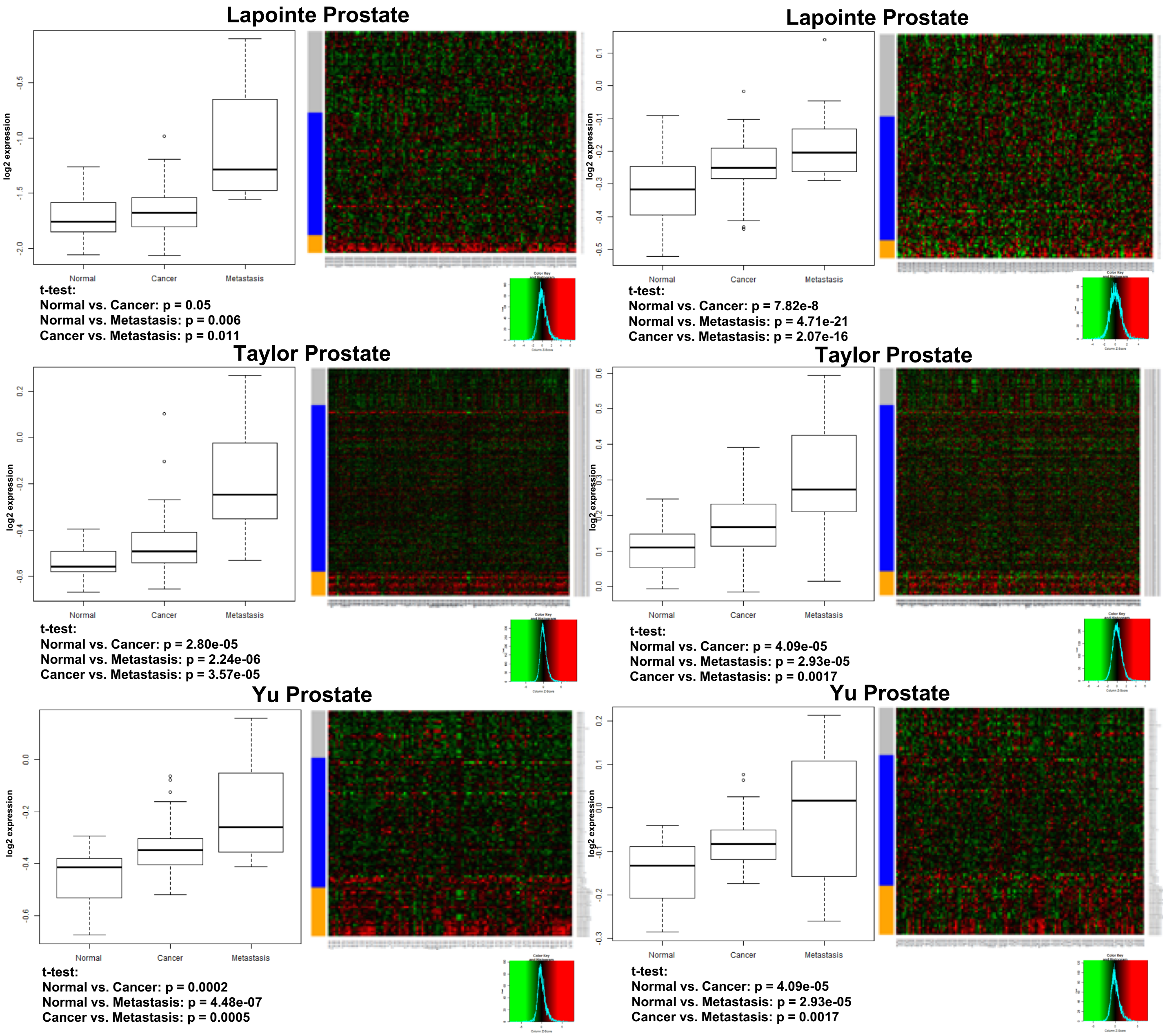

Appendix Figure S3. Multiple data sets demonstrate that the PARP-1 responsive transcriptome is elevated as a function of disease progression. The significantly PARPi downregulated gened in LNCaP (left) and C4-2 (right) were selected for analyses in the indicated prostate data sets utilizing Oncomine. Boxplot was generated using the mean expression of the PARPi down-regulated genes in the indicated data sets. Statistical significance determined by *t* test.

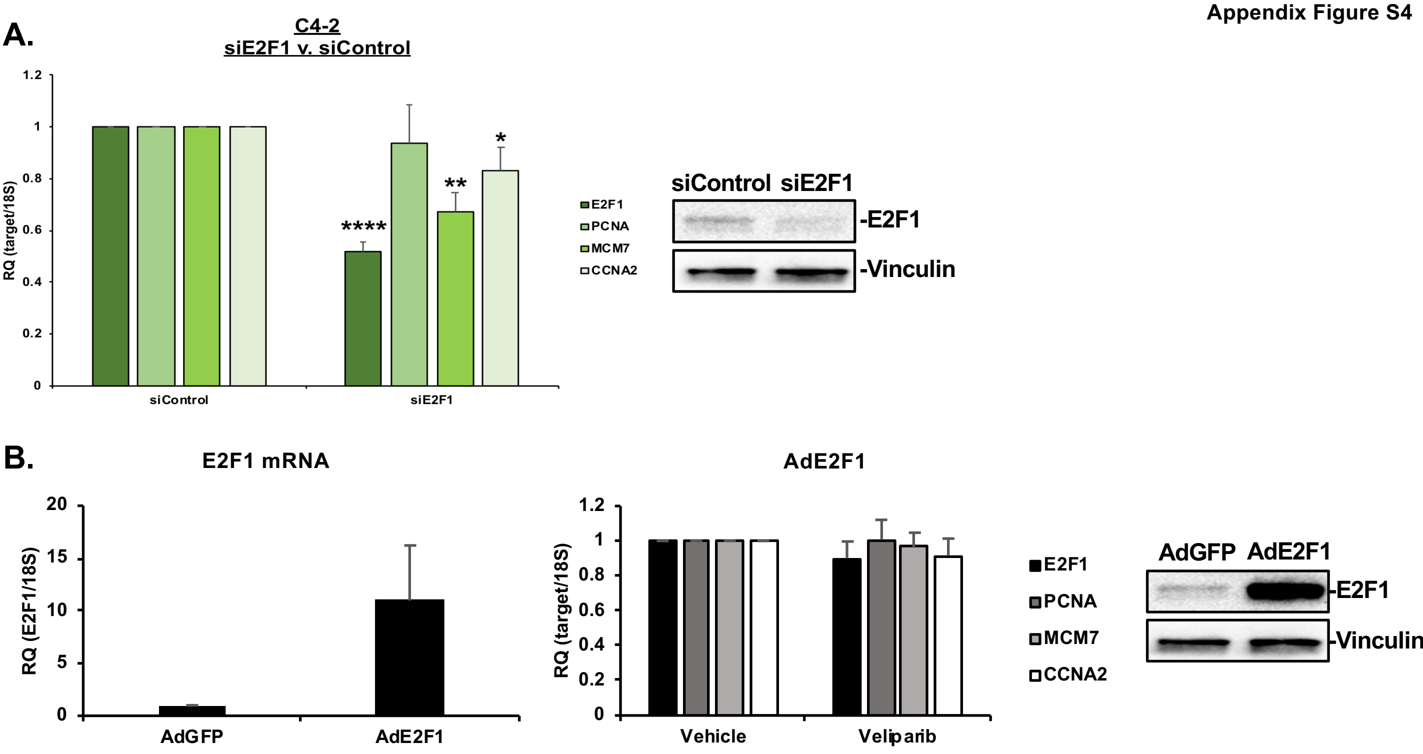

Appendix FigureS 4. PARP-1 control of E2F1-driven cell cycle gene expression is lost in the context of exogenous E2F1 expression. (A) C4-2 cells were transfected with either scrambled siRNA (siControl) or siRNA targeted to E2F1 (siE2F1). Data are depicted as mean +/- standard deviation of at least three independent biological experiments. Statistical significance was determine by Student's *t* test where *=p<0.05, **=p<0.01,****=p<0.0001. **(B)** C4-2 cells were infected with either a control GFP-encoding adenovirus (AdGFP) or an E2F1-encoding adenovirus (AdE2F1). Left: Exogenous E2F1 expression was validated via qPCR. Right: Cells infected as described were either treated with vehicle control or 2.5uM veliparib, and cell cycle gene expression was determined via qPCR.

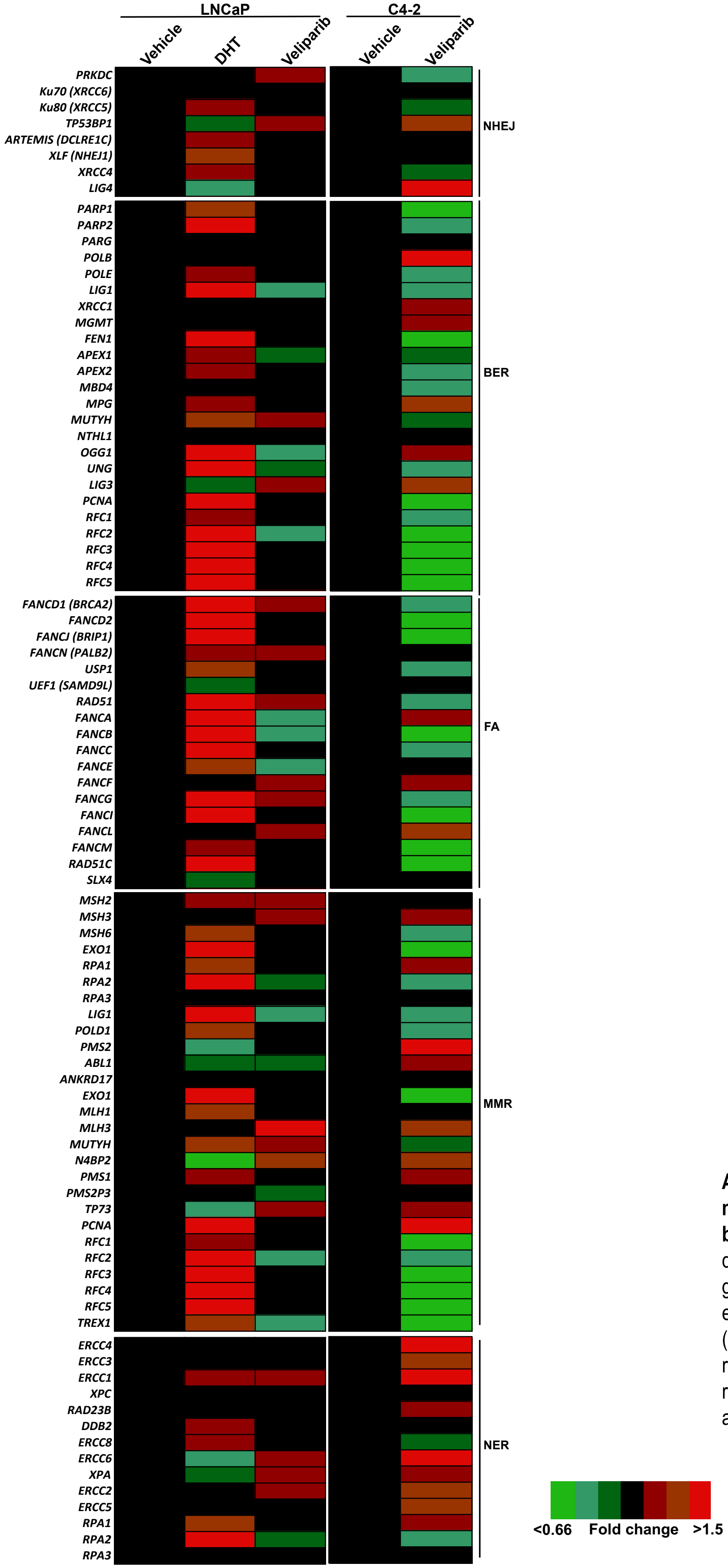

Appendix Figure S5. Expression of multiple DNA repair factors is effected by PARP inhibition. Data generated as described above in Figure 3 was used to generate heatmaps of non-homologous end-joining (NHEJ), base excision repair (BER), Fanconi Anemia (FA), mismatch repair (MMR), and nucleotide excision repair (NER) DNA repair gene expression after the indicated treatment regimens.

A.

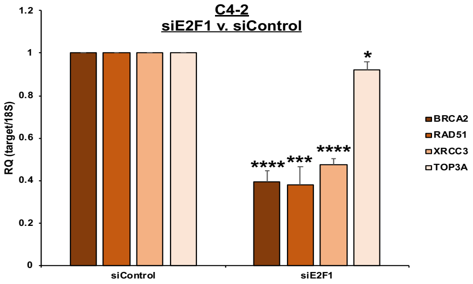

B.

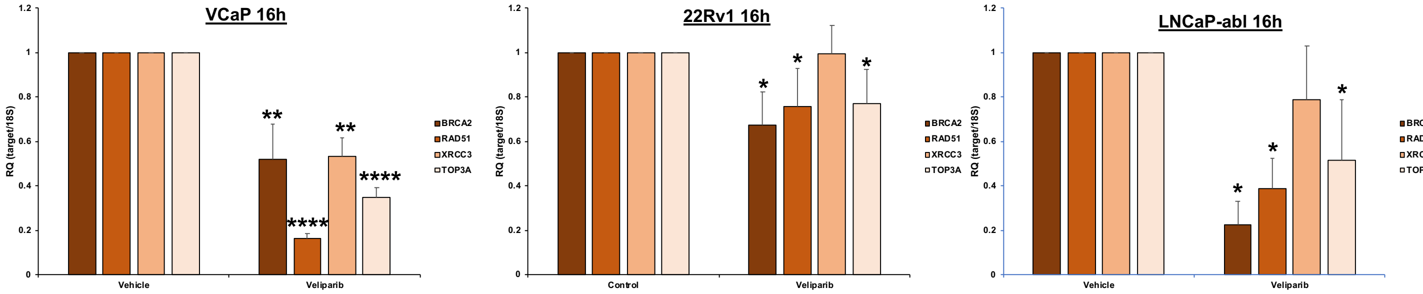

Appendix Figure S6. E2F1-driven HR expression is reduced by PARPi. (A) C4-2 cells were transfected with either scrambled siRNA (siControl) or siRNA targeted to E2F1 (siE2F1). Data are depicted as mean +/- standard deviation of at least three independent biological experiments. Statistical significance was determine by Student's t test where *=p<0.05, **=p<0.01,****=p<0.0001. (**B**) Indicated cell lines were treated as depicted in Figure 2. Data are depicted as mean +/- standard deviation of at least three independent biological experiments. Statistical significance was determine by Student's t test where *=p<0.05, **=p<0.01,****=p<0.0001.

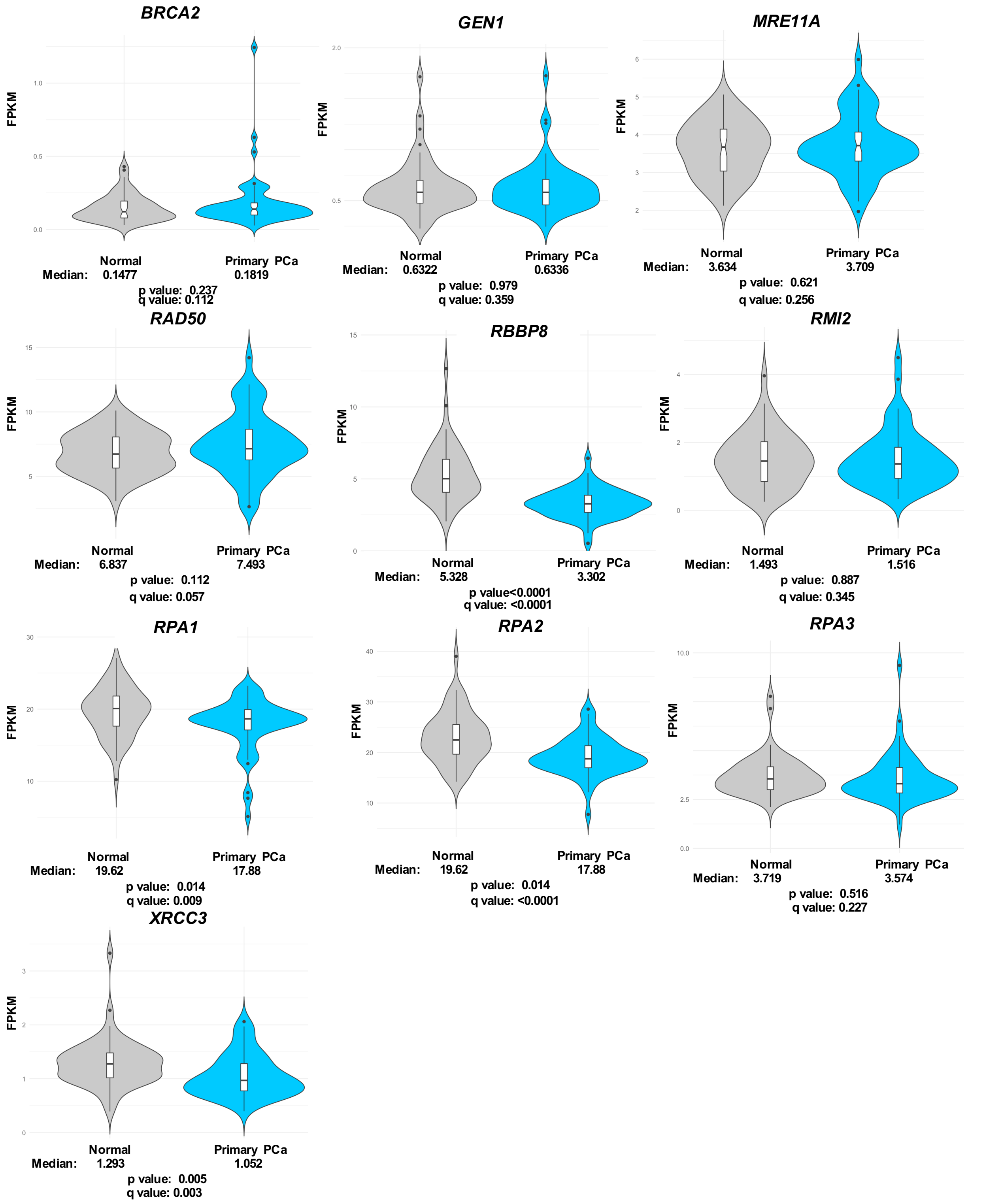

Appendix Figure S7. Expression levels of indicated HR pathway genes in primary PCa vs normal patient samples. Violin plots represent FPKM normalized counts obtained from matched tumor and normal RNA-Seq data from TCGA (n=52) with p-values generated using paired t-tests.

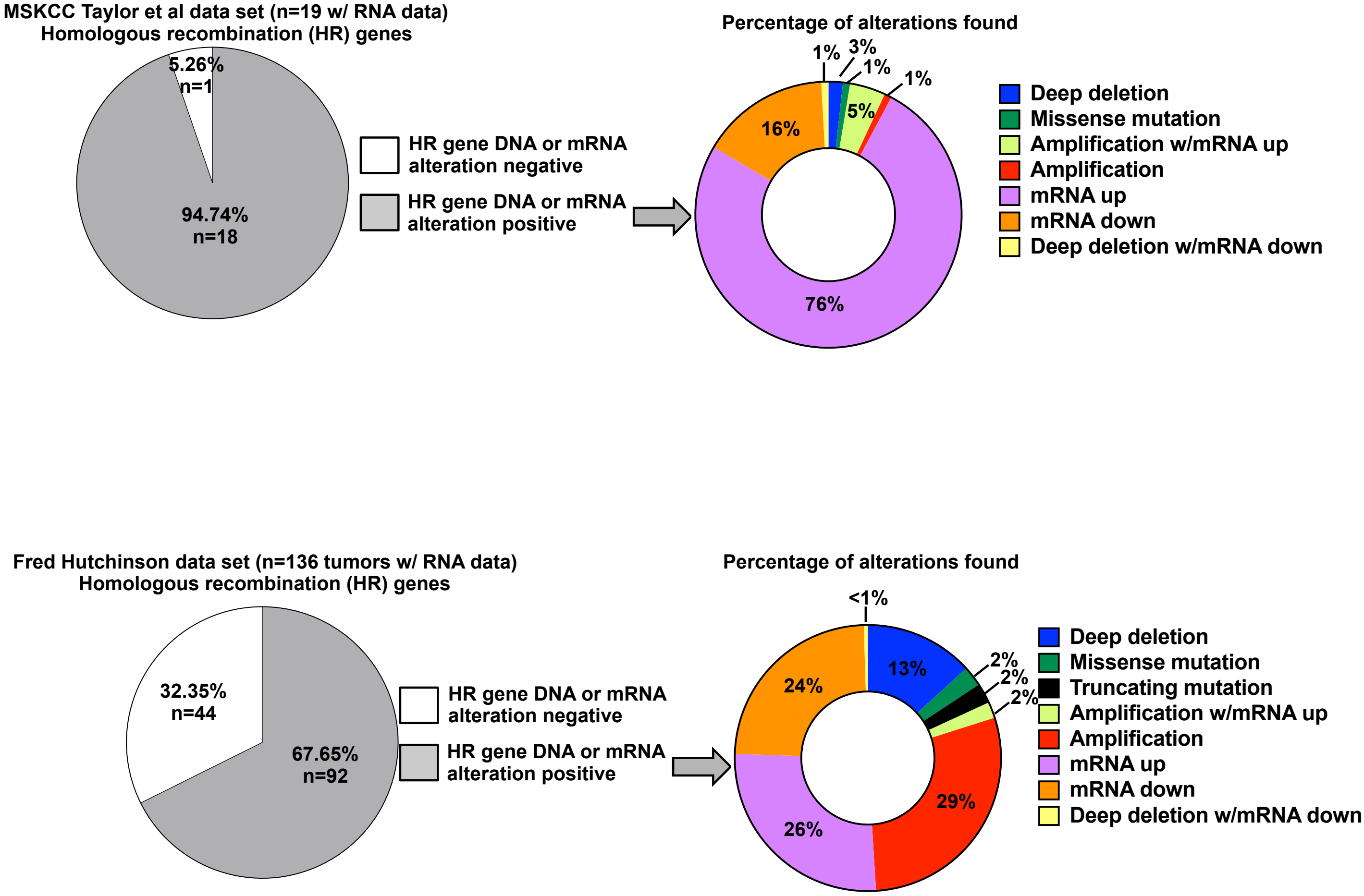

Appendix Figure S8. The most frequent type of HR gene alteration in PCa is mRNA up-regulation. The CBioportal was used to query the DNA and RNA HR gene alterations found in the MSKCC metastatic data set (top) and the Fred Hutchinson metastatic data set (bottom). Genes queried were *BRCA1*, *BRCA2*, *RAD51*, *MRE11A*, *RAD50*, *NBN*, *RBBP8*, *EXO1*, *RPA1*, *RPA2*, *RPA3*, *XRCC3*, *BLM*, *RMI1*, *RMI2*, *TOP3A*, *GEN1*, *SLX4*. Default settings were used.

Appendix Table S1

**mRNA primer sequences**		**ChIP primer sequences**	
RAD51 F	CAACCCATTTCACGGTTAGAGC	BRCA2 E2F1 F	CGAGCTTCTGAAACTAGGCG
RAD51 R	TTCTTTGGCGCATAGGCAACA	BRCA2 E2F1 R	AATCTGTCCCCTCACGCTTC
BRCA2 F	CACCTCTGGAGCGGACTTATT	RAD51 E2F1 F	CTTGCTCCAGGAATGCGAGT
BRCA2 R	GCTTTGTTGCAGCGTGTCTT	RAD51 E2F1 R	GACTATTAGCGGGGGCCCTA
XRCC3 F	GCCGATGTGGACACCTTGTTGG	TOP3A E2F1 F	AATCGGACTCCTTCTTGCGG
XRCC3 R	TGGTTGATGCACAGCACAGGGC	TOP3A E2F1 R	GTCCTCGTAGACTTCCGAGC
TOP3A F	GAGGCGGAGAGAAGGACTTT	E2F1 E2F1 F	CGGCGGTTCCTATTGGCTT
TOP3A R	GGTTGCAGCTCTGCCATTTTC	E2F1 E2F1 R	CCTGGTACCATCCGGACAAA
18S F	GCAATTATTCCCCATGAACG	Gene Desert F	GGGATGATGTGTGGGTTTTAC
18S R	GGCCTCACTAAACCATCCAA	Gene Desert R	CAATATCCAGCGAAAAGGAAGCT
E2F1 F	CTG GAC CTG GAA ACT GAC CAT		
E2F1 R	TTC ACA CCT TTT CCT GGA TGG C		
CYCLIN A2 F	CAG AAA ACC ATT GGT CCC TC		
CYCLIN A2 R	CAC TCA CTG GCT TTT CAT CTT C		
PCNA F	TGT GCA AAA GAC GGA GTG AA		
PCNA R	ACT CTA CAA CAA GGG GTA CAT C		
MCM7 F	CCTCGCAGCCAGTACACAA		
MCM7 R	GCCCCACCCTCTAAGGTCA		
**Antibodies**		**siRNA (GE Dharmacon)**	
PAR	Trevigen 4335-AMC-050	E2F1	L-003259-00-0005
γH2AX	Cell Signaling Technology 2577	Control	D-001810-10-50
PARP-1	Active Motif 39559		
E2F1	Bethyl Labs A300-766A		
RNA Polymerase II	Santa Cruz sc--899		
BRCA2	Santa Cruz sc-293185		
Vinculin	Sigma Aldrich V9264-200UL		
53BP1	Novus Biologicals NB100-304		
RAD51	Santa Cruz SC-8349		
Lamin B	Santa Cruz sc-6217		
Histone H4	Millipore 07-108		
RB	BD Parmingen 554136		
CBP	Abcam ab10490		
