# Supplementary material for: PARP‐1 regulates DNA repair factor availability
Source: EMBO Mol Med. 2018 Nov 22;10(12):e8816. doi: 10.15252/emmm.201708816 (PMC6284389; doi:10.15252/emmm.201708816)
Supplement: Supplementary file 4 — Source Data for Figure 1 [file EMMM-10-e8816-s003.pdf]

Microscopy images
Related to Figure 1A

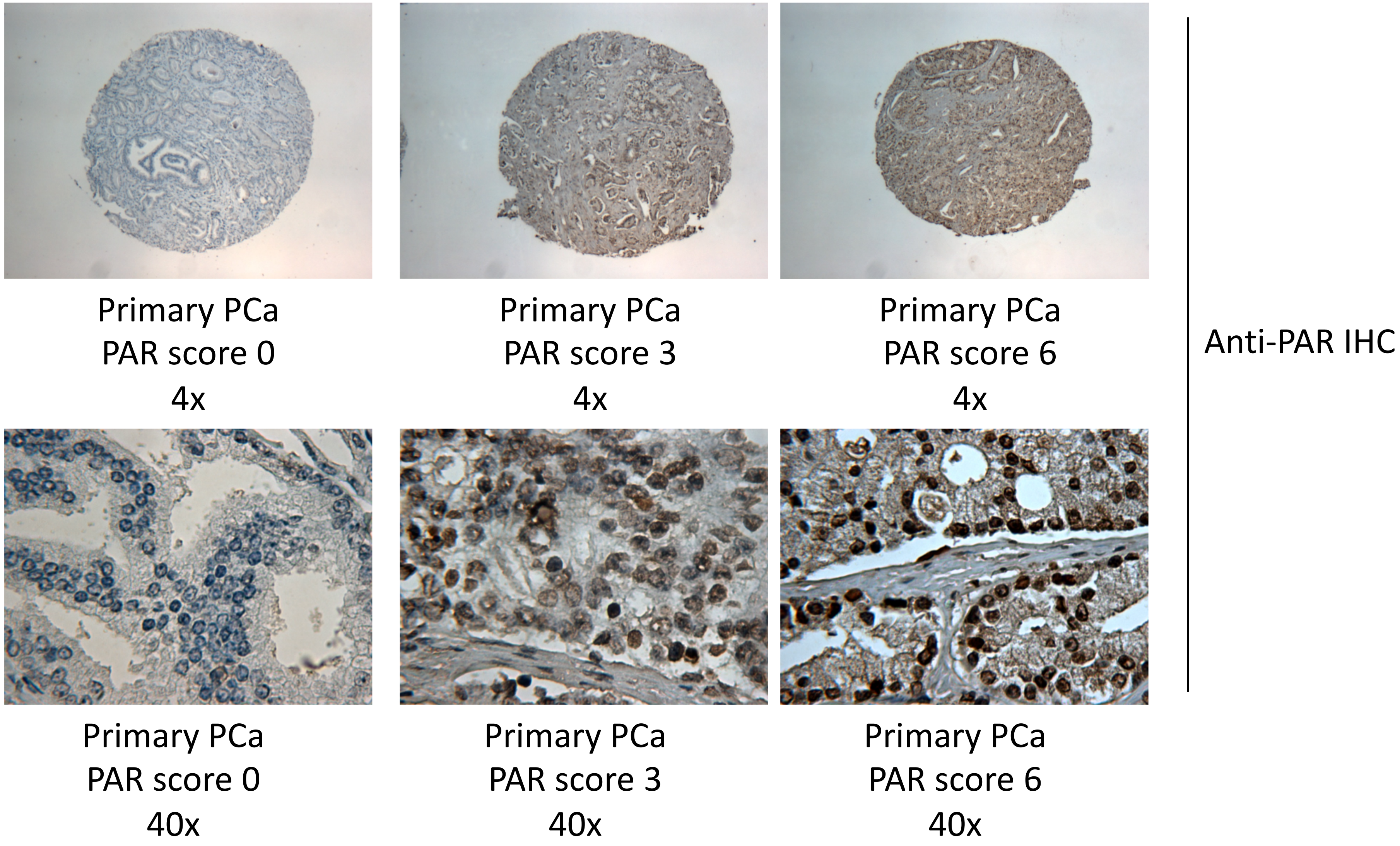

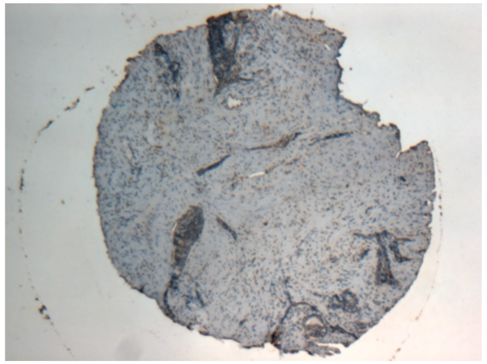

mCRPC
PAR score 3
4x

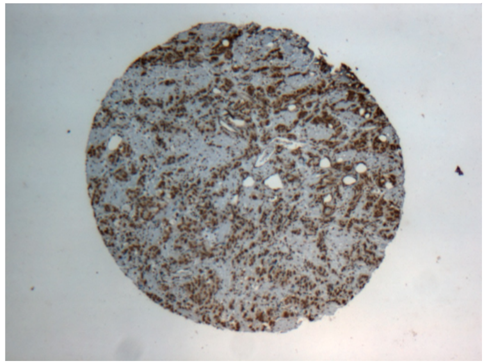

mCRPC
PAR score 6
4x

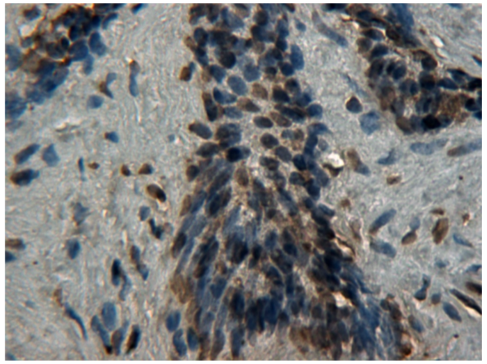

Primary PCa
PAR score 3
40x

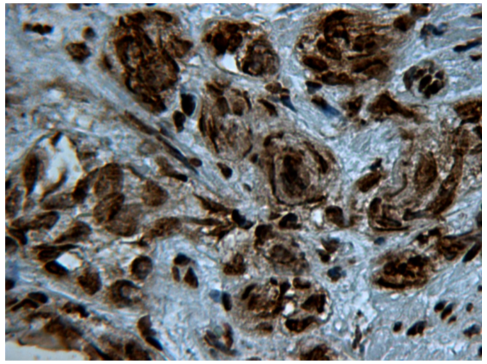

Primary PCa
PAR score 6
40x

Anti-PAR IHC
