# Supplementary figures and images for: PARP‐1 regulates DNA repair factor availability
Source: EMBO Mol Med. 2018 Nov 22;10(12):e8816. doi: 10.15252/emmm.201708816 (PMC6284389; doi:10.15252/emmm.201708816)

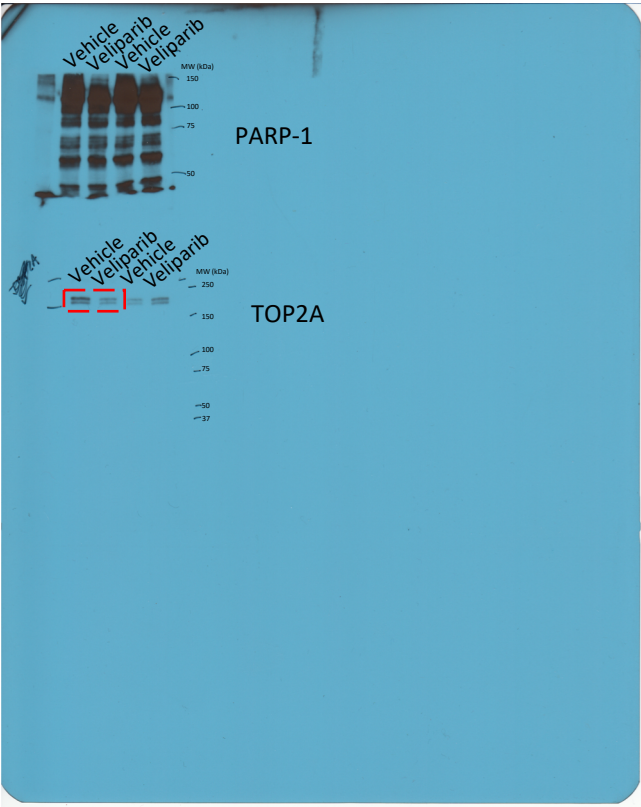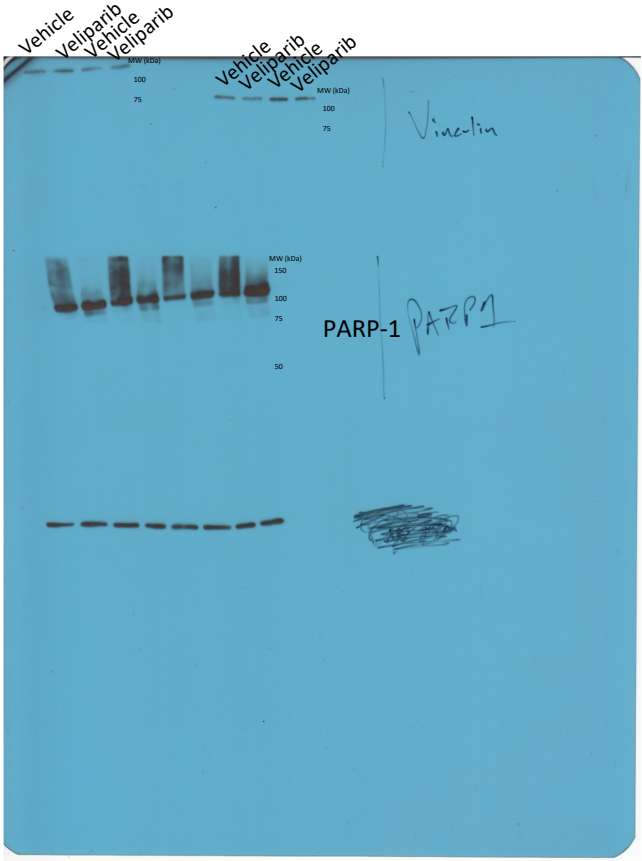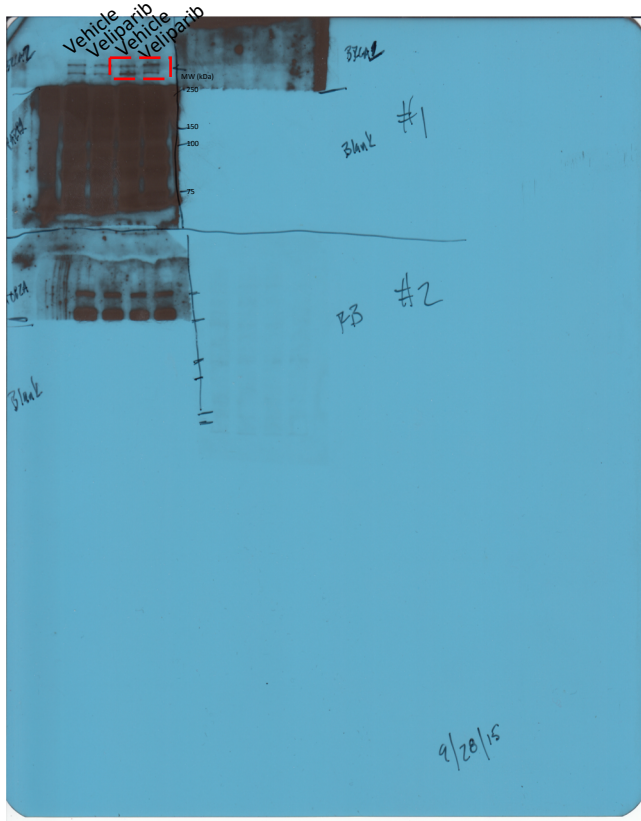

Supplement: Supplementary file 5 — Source Data for Figure 5 [file EMMM-10-e8816-s004.pdf]
